# Supplementary material for: Photocatalytic Oxidation of Propane Using Hydrothermally Prepared Anatase-Brookite-Rutile TiO2 Samples. An In Situ DRIFTS Study
Source: Nanomaterials (Basel). 2020 Jul 4;10(7):1314. doi: 10.3390/nano10071314 (PMC7407931; doi:10.3390/nano10071314)
Supplement: Supplementary file 1 [file nanomaterials-10-01314-s001.pdf]

nanomaterials

MDPI

Supplementary Materials

Photocatalytic Oxidation of Propane Using Hydrothermally Prepared Anatase-Brookite-Rutile TiO2 Samples. An *In Situ* DRIFTS Study

Laura Cano-Casanova [1,*], Bastian Mei [2], Guido Mul [2], María Ángeles Lillo-Ródenas [1] and María del Carmen Román-Martínez [1]

[1] MCMA Group, Department of Inorganic Chemistry and Materials Institute (IUMA). Faculty of Sciences, University of Alicante, Ap. 99, E-03080 Alicante, Spain; mlillo@ua.es (M.A.L.-R.); mcroman@ua.es (M.C.R.-M.)

[2] PhotoCatalytic Synthesis Group, MESA + Institute for Nanotechnology, Faculty of Science and Technology, University of Twente, 7500 AE Enschede, The Netherlands; b.t.mei@utwente.nl (B.M.); g.mul@utwente.nl (G.M.)

* Correspondence: laura.cano@ua.es; Tel.: +3496-5903-545; Fax: +3496-5903-454

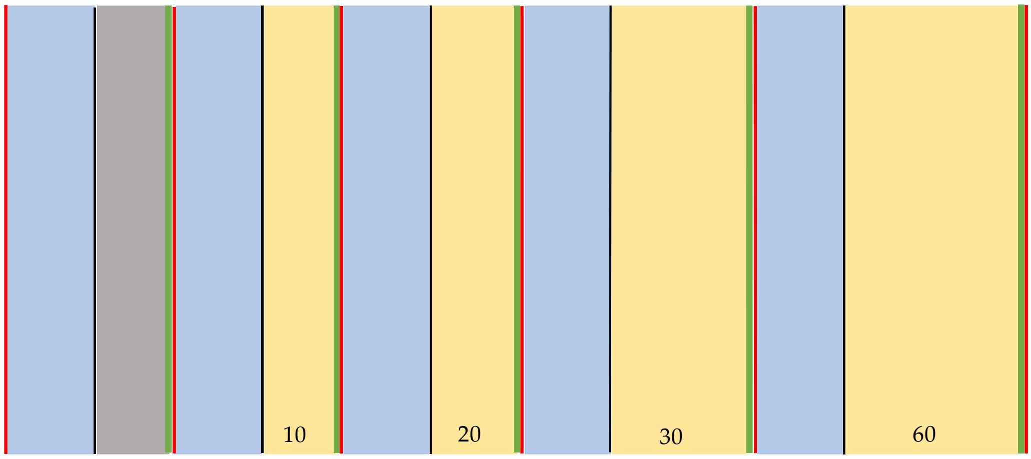

Gas flow through the reaction chamber 0.15 ml/min propane, 5.85 ml/min O_2 and 24 ml/min N_2 (21 minutes).
Closure of reaction chamber.
Propane in reaction chamber in dark conditions (10 minutes).
Opening of reaction chamber and content sweping with He flow (10 seconds).
Standby time (5 seconds).
Propane in reaction chamber under illumination (10, 20, 30 and 60 minutes).

Figure S1. Steps involved in each experiment, probing oxidation of propane.

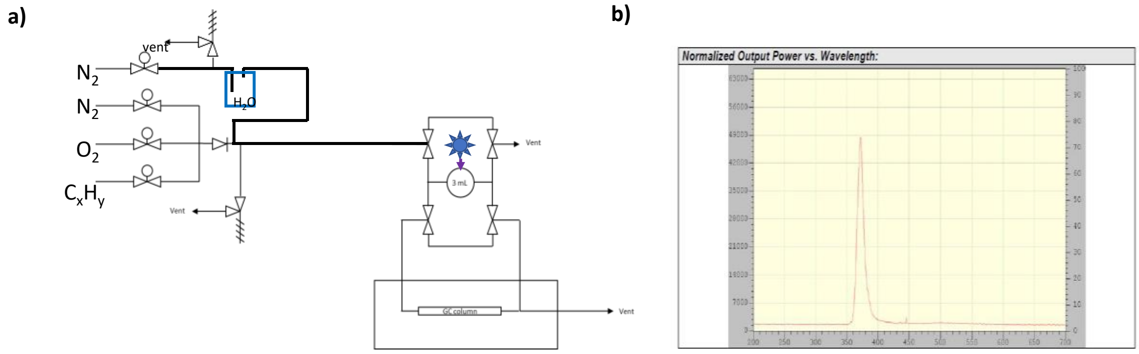

Scheme S1. (**a**) Flowsheet of the batch reactor connected to a gas chromatograph and (**b**) Irradiation spectrum of 375 nm LED.

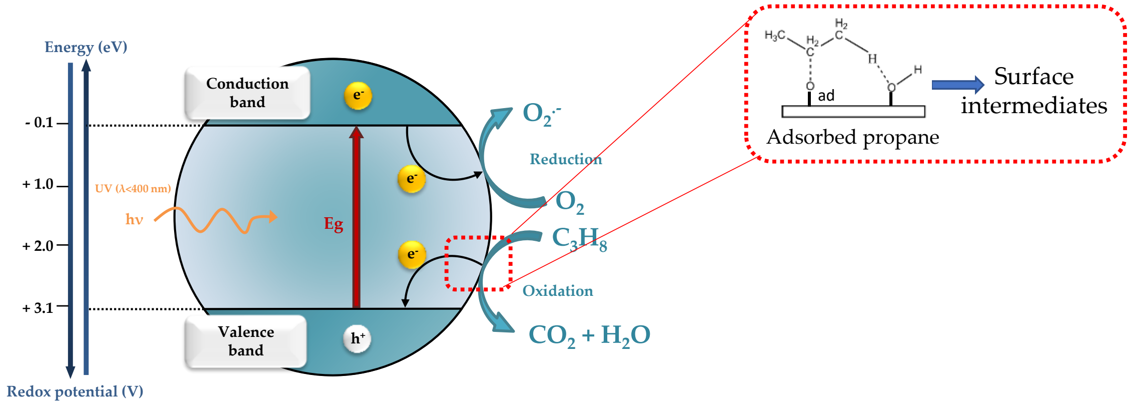

Scheme S2. Schematic picture of the photocatalytic process on a TiO_2 particle.

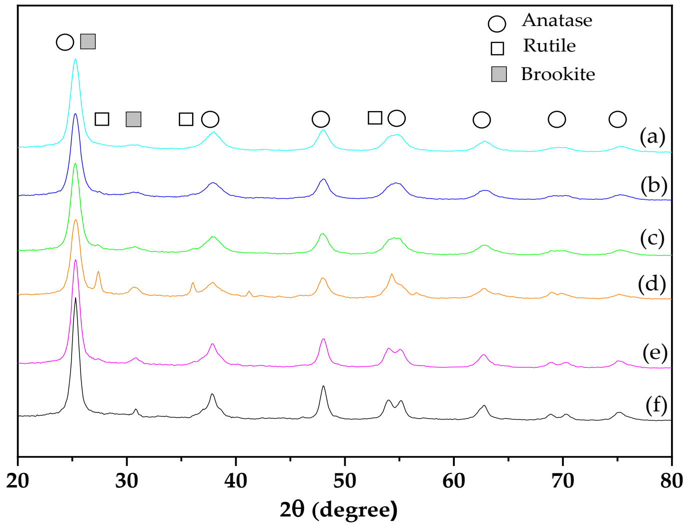

Figure S2. XRD patterns of TiO_2 samples prepared using HCl solutions of different concentration: (**a**) TiO_2-0.5M, (**b**) TiO_2-0.8M, (**c**) TiO_2-1M, (**d**) TiO_2-3M, (**e**) TiO_2-7M and (**f**) TiO_2-12M. Re-drawn from reference [10].

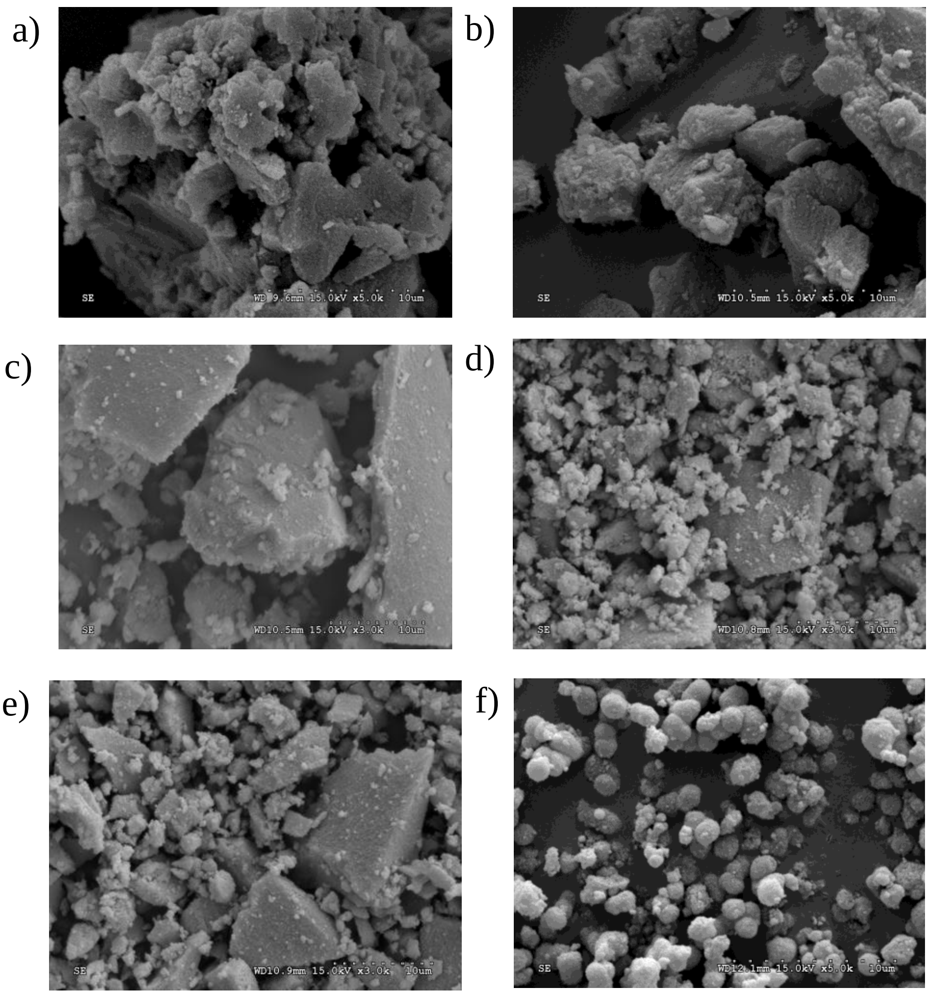

Figure S3. SEM images (with scale bar of 10 μm) of samples: (**a**) TiO_2-0.5M, (**b**) TiO_2-0.8M, (**c**) TiO_2-1M, (**d**) TiO_2-3M, (**e**) TiO_2-7M and (**f**) TiO_2-12M.

Table S1. "Propane dark" and propane adsorbed in dark conditions.

Sample	"Propane Dark" (ppmv)	Propane Adsorbed in Dark* (mmol/g)
TiO_2-0.5M	3111	0.57
TiO_2-0.8M	3969	0.30
TiO_2-1M	4068	0.26
TiO_2-3M	4539	0.11
TiO_2-7M	4085	0.26
TiO_2-12M	3053	0.59
P25	4675	0.07

* Propane adsorbed in dark= initial propane concentration minus "propane dark" (expressed as mmol/g).

Table S2. Propane concentration (in ppm) in the exhaust gas of the reactor in blank experiments.

Blank Experiment*	Propane Dark (ppm)
1	4893
2	4884

* Glass support without photocatalyst.

Table S3. Humidity, quantity of various OH-groups (in weight percentages), and density of OH-groups (OH_T/S_{BET}).

Sample	Humidity (%)	OH_{weak} (%)	OH_{strong} (%)	OH_{total} (%)	OH_T Density x 10^{-18} (OH Groups/m²)
TiO_2-0.5M	1.40	1.48	0.88	2.36	6.19
TiO_2-0.8M	1.26	1.40	0.97	2.37	6.27
TiO_2-1M	1.27	1.34	0.72	2.06	6.29
TiO_2-3M	1.27	1.23	0.69	1.92	5.81
TiO_2-7M	0.94	1.10	0.67	1.77	5.55
TiO_2-12M	1.03	1.12	0.76	1.88	6.05
P25	0.61	0.76	0.51	1.11	10.21

[a] Determined by the weight loss in the interval 30–120 °C.
[b] Determined by the weight loss in the interval 120–300 °C.
[c] Determined by the weight loss in the interval 300–600 °C.
[d] Sum of OH_{weak} and OH_{strong}
[e] OH total as amount of OH groups divided by surface area.

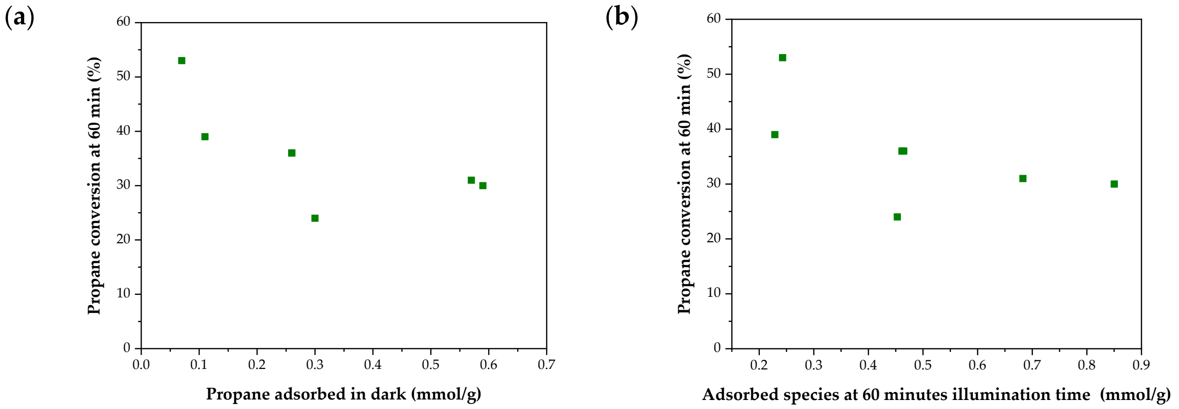

Figure S4. (**a**) Propane conversion (%) at 60 min vs propane adsorbed (mmol/g) in absence of light. (**b**) Propane conversion (%) at 60 min vs the quantity of adsorbed species (mmol/g, equation 5) after 60 min of illumination.

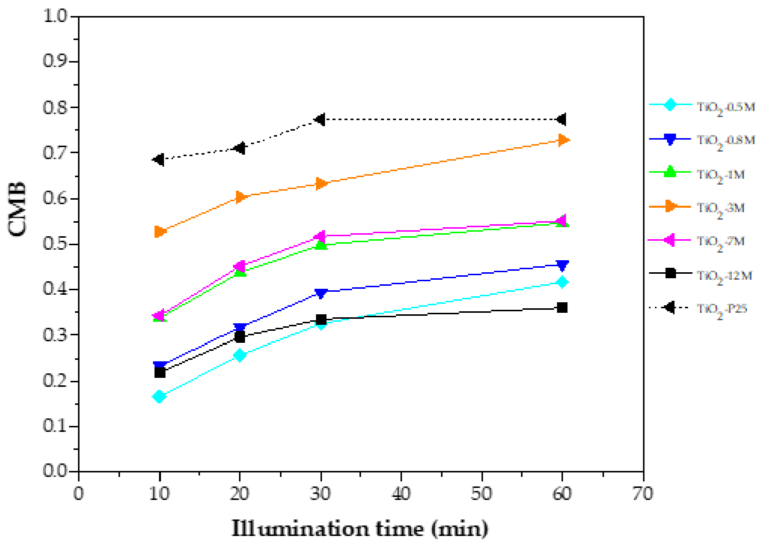

Figure S5. Carbon mass balance (CMB) versus irradiation time for the TiO_2 samples.

The CMB at each illumination time, is calculated as:

$$CMB = \frac{1}{3} \times \frac{CO_2\,produced}{C_3H_8\,removed} \quad (1)$$

A value of CMB = 1 is indicative of negligible propane adsorption and, indeed, in samples with low amount of adsorbed propane the CMB is closer to 1.

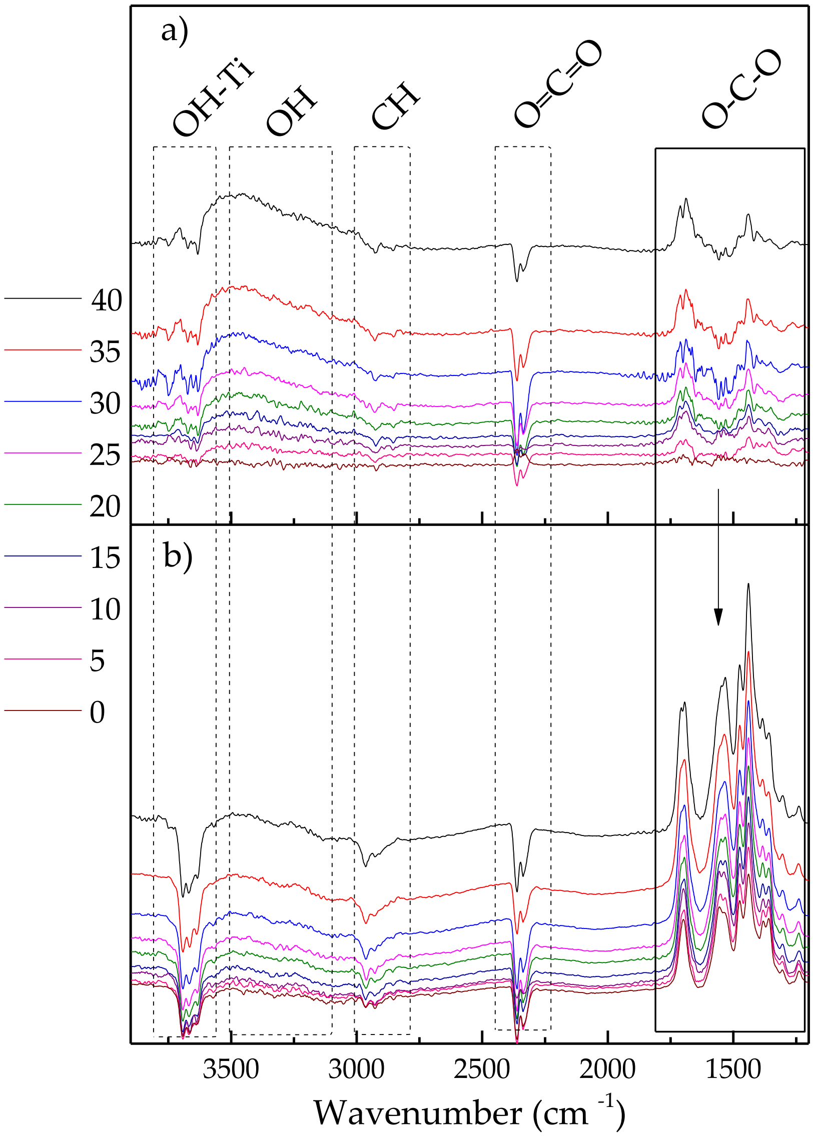

Figure S6. DRIFTS spectra of the TiO_2-12M sample measured every 5 minutes under irradiation, for a total irradiation time of 40 minutes: (**a**) without propane and (**b**) with propane (t = 0 corresponds to dark).

Table S4. Assignment of bands in the frequency range 1200–3700 cm^{-1} for Figure S3.

IR region (cm^{-1})	Species	Reference
3600–3700	OH groups bound to single Ti atoms	[27]
• 3693 and 3632 stretching modes of free -OH groups on Ti^{4+}anatase		[27,28]
• 3665 bridging hydroxyls. There is no evidence of Ti^{3+}-OH bands (that would have appeared at 3617 cm^{-1} [29]).		[27,28]
3350–3450	O-H stretching of physisorbed water and hydrogen-bonded hydroxyl groups	[30]
2800–3000	C-H species	[31].
2300–2400	CO_2 bending modes	[32]
1200–1800	R–CO_2^- species (formate, carbonate)	[5,21,24,33]

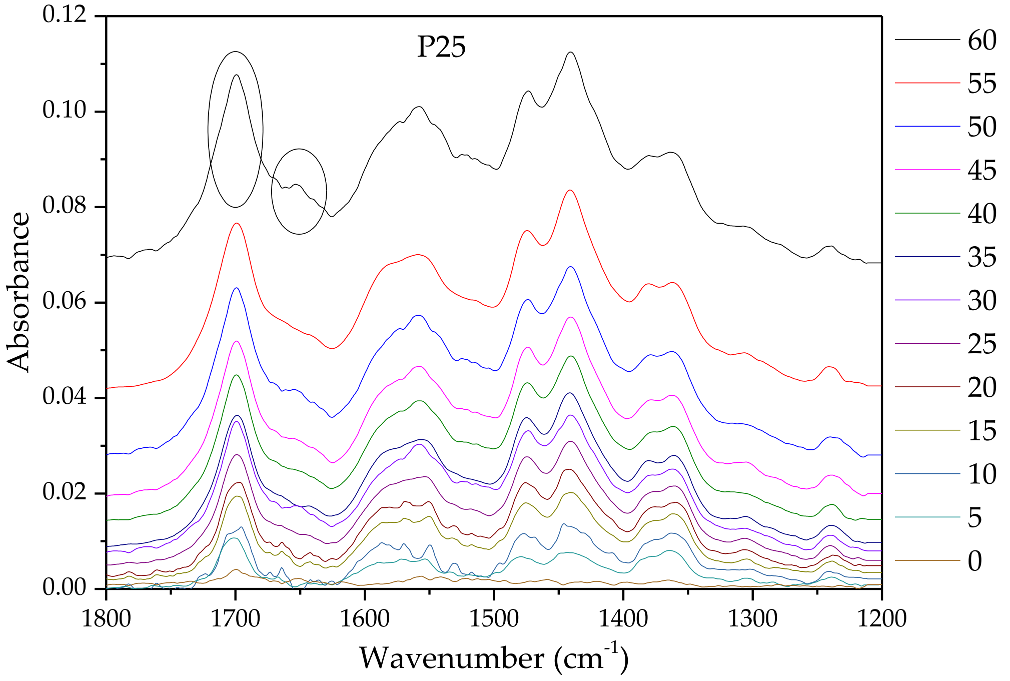

Figure S7. DRIFTS spectra of sample P25. Spectra were recorded every 5 minutes under irradiation in a propane atmosphere, for a total irradiation time of 60 min (see irradiation time colors, from 0 to 60 min). The spectrum at t = 0 was recorded in dark.

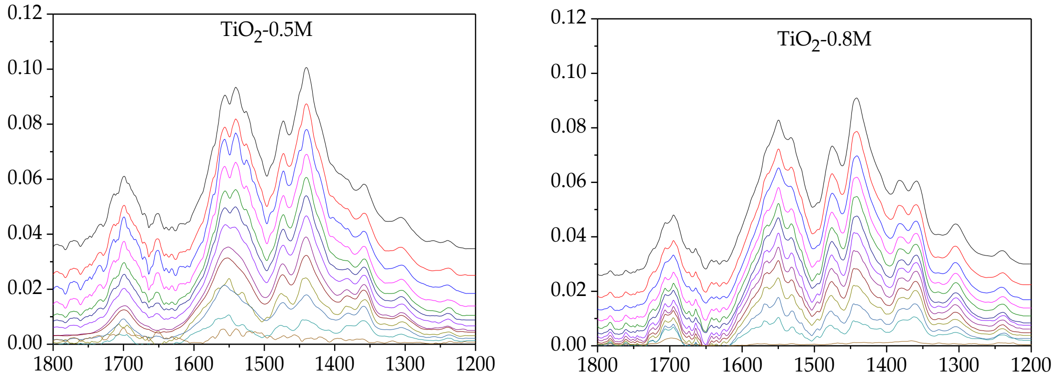

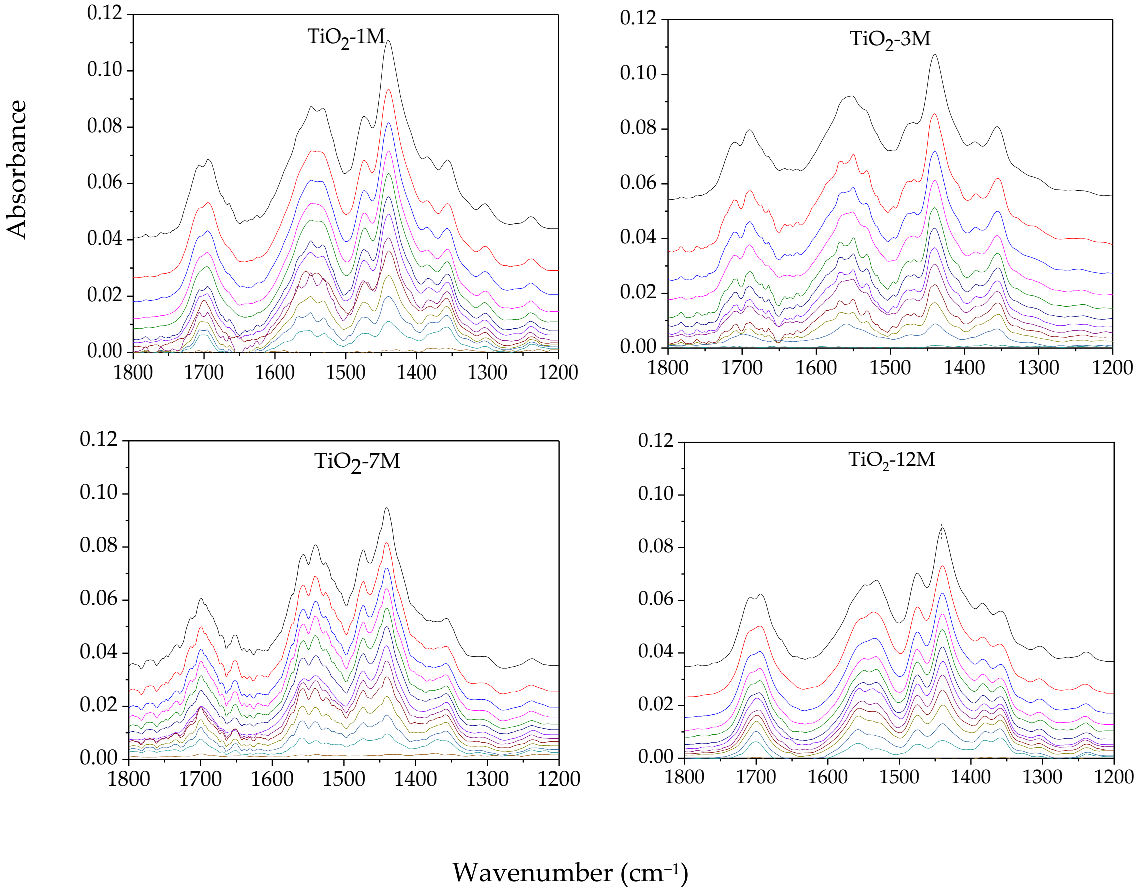

Figure S8. DRIFTS spectra of the series of TiO_2-XM samples. Spectra were recorded every 5 minutes under irradiation in a propane atmosphere, for a total irradiation time of 60 min (color codes are the same as in Figure S4, from 0 to 60 min). The spectrum at t = 0 was recorded in the absence of light.

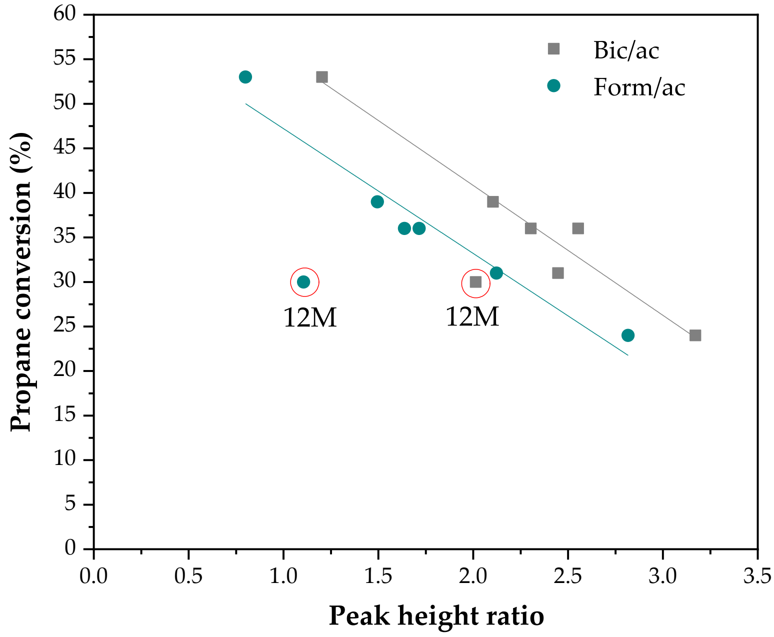

Figure S9. Propane conversion (60 min) vs peak height ratios (bic/ac and form/ac, height of peak at 1556 cm^{-1}/ height of peak at 1690 cm^{-1} and height of peak at 1438 cm^{-1}/ height of peak at 1690 cm^{-1}, respectively).
